# Edge restenosis: impact of low dose irradiation on cell proliferation and ICAM-1 expression

**DOI:** 10.1186/1471-2261-6-32

**Published:** 2006-07-07

**Authors:** Rainer Voisard, Jochen Höb, Regine Baur, Tina Herter, Andreas Hannekum, Vinzenz Hombach

**Affiliations:** 1 Department of Internal Medicine II – Cardiology, University of Ulm, Robert-Koch-Straße 8, D-89081 Ulm, Germany; 2 Department of Cardiac Surgery, University of Ulm, Steinhövelstraße 9, 89070 Ulm, Germany

## Abstract

**Background:**

Low dose irradiation (LDI) of uninjured segments is the consequence of the suggestion of many authors to extend the irradiation area in vascular brachytherapy to minimize the edge effect. Atherosclerosis is a general disease and the uninjured segment close to the intervention area is often atherosclerotic as well, consisting of neointimal smooth muscle cells (SMC) and quiescent monocytes (MC). The current study imitates this complex situation in vitro and investigates the effect of LDI on proliferation of SMC and expression of intercellular adhesion molecule-1 (ICAM-1) in MC.

**Methods:**

Plaque tissue from advanced primary stenosing lesions of human coronary arteries (9 patients, age: 61 ± 7 years) was extracted by local or extensive thrombendarterectomy. SMC were isolated and identified by positive reaction with smooth muscle α-actin. MC were isolated from buffy coat leukocytes using the MACS cell isolation kit. For identification of MC flow-cytometry analysis of FITC-conjugated CD68 and CD14 (FACScan) was applied. SMC and MC were irradiated using megavoltage photon irradiation (CLINAC2300 C/D, VARIAN, USA) of 6 mV at a focus-surface distance of 100 cm and a dose rate of 6 Gy min^-1 ^with single doses of 1 Gy, 4 Gy, and 10 Gy. The effect on proliferation of SMC was analysed at day 10, 15, and 20. Secondly, total RNA of MC was isolated 1 h, 2 h, 3 h, and 4 h after irradiation and 5 μg of RNA was used in standard Northern blot analysis with ICAM-1 cDNA-probes.

**Results:**

Both inhibitory and stimulatory effects were detected after irradiation of SMC with a dose of 1 Gy. At day 10 and 15 a significant antiproliferative effect was found; at day 20 after irradiation cell proliferation was significantly stimulated. Irradiation with 4 Gy and 10 Gy caused dose dependent inhibitory effects at day 10, 15, and 20. Expression of ICAM-1 in human MC was neihter inhibited nor stimulated by LDI.

**Conclusion:**

Thus, the stimulatory effect of LDI on SMC proliferation at day 20 days after irradiation may be the in vitro equivalent of a beginning edge effect. Extending the irradiation area in vascular brachytherapy in vivo may therefore merely postpone and not inhibit the edge effect. The data do not indicate that expression of ICAM-1 in quiescent MC is involved in the process.

## Background

The convincing results of drug elutings stents (DES) have ended the era of vascular brachytherapy as treatment strategy against restenosis. Before that time both β and γ radiation had been used as promising approach to inhibit restenosis after percutaneous coronary interventions [1-4]. However, even before the use of DES, angiographic follow-up studies after irradiation reported that although restenosis was inhibited in the middle of the irradiation area increased restenosis was detected at both ends of the irradiation field. This effect was called edge effect or candy wrapper effect [3-5].

The cause of the edge effect is not completely understood. Current explanations are the stimulation of tissue proliferation or extracellular matrix by low dose irradiation (LDI), by mechanical injury, or by a combination of both [6]. Many authors suggest therefore to minimize the edge effect by extending the irradiation area from each end of the stent [7,8]. However if the radiation margins are extended, uninjured segments will be irradiated with low radiation doses. It is not yet entirely clear whether the subtherapeutic levels of radiation in uninjured segements will not cause an inhibitory and later stimulatory effect [5,9,10]. A stimulation of neointimal proliferation after LDI of uninjured segments has already been reported by Powers et al. [9].

In the pathophysiology of the typical restenosis process an increased proliferative activity of human coronary smooth muscle cells (SMC) is of central importance [11]. Recently it has been demonstrated that the stimulation of the transcriptional programs governing neointimal formation in humans are very complex [12]. Some authors are advocating that the key cells responsible for restenosis are monocytes/macrophages, the same cells that initiate atherosclerotic plaques [review, [13]]. Intercellular adhesion molecule-1 (ICAM-1) is a cell surface molecule that mediates adhesion processes involving leukocytes and other cell types [14].

The uninjured region close to the intervention area is often atherosclerotic as well, consisting of smooth muscle cells (**h**uman **p**laque **SMC **= HPSMC) and quiescent monocytes (MC). If irradiation is extended on both sides of the injured area in order to prevent the edge effect, HPSMC and quiescent MC will be treated with LDI. The present study investigates the effect of LDI on proliferation of coronary HPSMC and ICAM-expression in quiescent MC.

## Methods

### Cell isolation, cultivation and identification

Plaque tissue from advanced primary stenosing lesions of human coronary arteries (9 patients, age: 61 ± 7 years) was extracted by local or extensive thrombendarterectomy. HPSMC were isolated by enzymatic disaggregation and identified by positive reaction with smooth muscle α-actin, as described [11]. Isolated cells were cultured in a mixture of Waymouth's MB 752/1 medium and Ham F12 nutrient mixture (1:1; v/v) supplemented with 15% fetal calf serum and standard amounts of antibiotics. Pooled cells of donors from both sexes were used for cell proliferation studies.

MC were isolated from buffy coat leukocytes of healthy donors using the MACS cell isolation kit (Milteny Biotec, Bergisch Gladbach, Germany). Identification of MC was carried out by flow-cytometry analysis of FITC-conjugated CD68 and CD14 (FACScan). The percentage of CD68 and CD14 positive cells was analysed for each single isolation and the average was calculated.

All cell culture studies were carried out with the permission of the Ethical Committee of the University of Ulm, D.

### Irradiation procedure

HPSMC in passages 3–5 were seeded in a density of 3 – 5 × 10^3 ^cells × cm^-2 ^in cell culture flasks (12.5 cm^2^). 24 h after seeding the culture medium was renewed and the number of adherent cells was analyzed in a cell counter (CASY TTC, Schärfe System, Reutlingen, D).

1 × 10^7 ^MC were seeded and cultured in cell culture dishes (75 cm^2^, Becton Dickinson, Heidelberg, Germany) for 40 min. at 37°C in RPMI supplemented with 10% fetal calf serum (fcs), before irradiation was started.

HPSMC and MC were irradiated 24 h respectively 40 min after seeding. Cells were irradiated using megavoltage photon irradiation (CLINAC2300 C/D, VARIAN, USA) of 6 mV at a focus-surface distance of 100 cm and a dose rate of 6 Gy min^-1 ^with single doses of 1 Gy, 4 Gy, and 10 Gy. Sham-irradiated cultures of SMC and MC were kept at room temperature in the X-ray control room while the other samples were irradiated.

### Measurement of cell proliferation

The effect of low dose irradiation on HPSMC proliferation was analysed at day 10, 15, and day 20 after irradiation. Culture medium and agents were renewed every second or third day. Cell number of HPSMC was calculated as relative cell number in comparison to untreated controls. Taking into account that not all HPSMC could be successully cultured, cell numbers of controls without irradiation were calculated as:

Total cell number at day X – number of attached cells at day 1 after seeding = 100%.

### RNA extraction and Northern Blot analysis

MC RNA extraction and Northern blot studies was carried out 1 h, 2 h, 3 h, and 4 h after low dose irradiation. For Northern blot studies total RNA was isolated with RNeasy Mini Kit^® ^(Quiagen, Hilden, Germany), and 5 μg of RNA was used in standard Northern blot analysis by using cDNA probes against ICAM-1 and housekeeping genes GAPDH. The cDNA probes were labeled with ^32^P (Rediprime II-Kit, Amersham, Pharmacia, Braunschweig, D). Hybridization was carried out overnight at 42°C. Phosphorimaging was used to detect the relative band density of ICAM-1 mRNA in comparison with untreated controls. Experiments were performed in triplicate, as control GAPDH was used.

### Statistical analysis

Data of proliferation studies and relative band densities of expression of ICAM-1 mRNA are presented as mean ± S.D. Statistical significance of differences between controls and irradiated cells was determined by paired Student's t-test. Statistical significance was accepted for p < 0.05.

## Results

### Identification of HPSMC and MC

**Figure 1:**
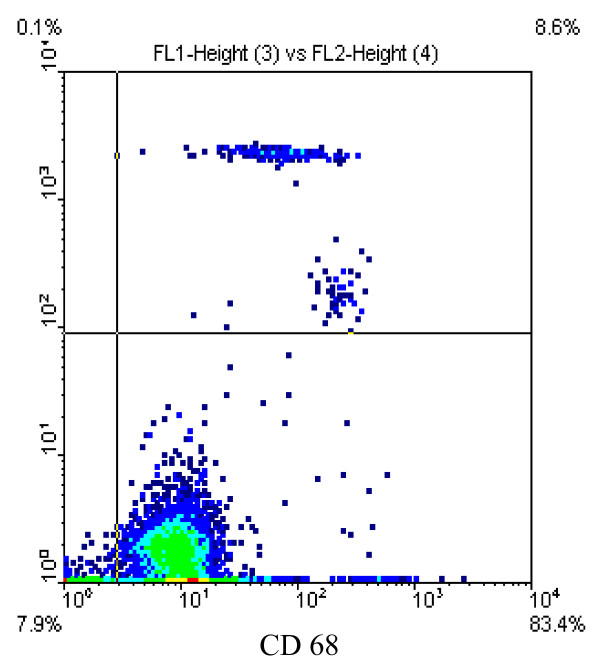
Isolation of monocytes (MC) using the MACS-Cell-Isolation-Kit. FACS-analysis with CD68-FITC demonstrating a purity of MC preparations of 80.6% (CD68).

HPSMC were identified by positive reaction with antibodies against smooth muscle α-actin and the typical "hill and valley" growth pattern. For identification of MC flow cytometry analysis was used. Average prurity of MC preparations were 80.6 ± 2.2% (CD68, Fig. 1) and 82 ± 5.5% (CD14).

### Effect of irradiation on proliferation of HPSMC

**Figure 2:**
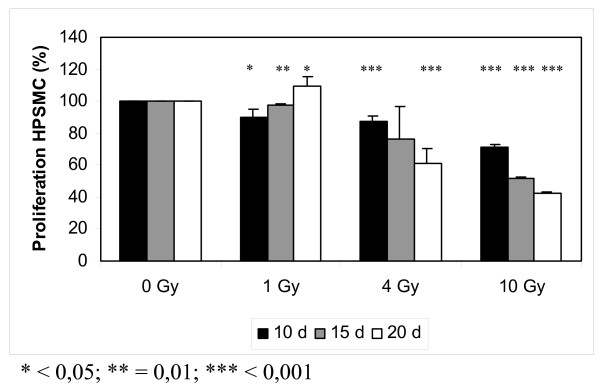
Effect of low dose irradiation (1 Gy, 4 Gy, and 10 Gy) on proliferation of smooth muscle cells from human coronary plaque material (HPSMC). The data indicate that low dose irradiation with 1 Gy inhibits proliferation of HPSMC in the short run (day 10) but stimulates HPSMC proliferation in the long run (day 20).

Proliferation of coronary HPSMC was analyzed at day 10, day 15, and day 20 after low dose irradiation (1 Gy, 4 Gy, and 10 Gy; Fig. 2).

After irradiation with 1 Gy both inhibitory and stimulatory effects were observed. 10 days and 15 days after irradiation a small inhibitory effect by 10% and 2.6% was detected (p < 0.05 and p = 0.01). 20 days after irradiation a small stimualtion of cell proliferation by 9.3% was seen (p < 0.05).

After irradiation with 4 Gy a time dependent inhibition of cell proliferation was found. 10 days, 15 days, and 20 days after irradiation cell proliferation was inhibited by 13.3% (p < 0.001), 23.3% (n.s.), and 39.2% (p < 0.001).

Irradiation with 10 Gy caused time dependent inhibition of cell proliferation. 10 days, 15 days, and 20 days after irradiation cell proliferation was inhibited by 28.7% (p < 0.001), 48.4% (p < 0.001), and 57.9% (p < 0.001).

### Effect of irradiation on ICAM-1 mRNA levels of monocytes

**Figure 3:**
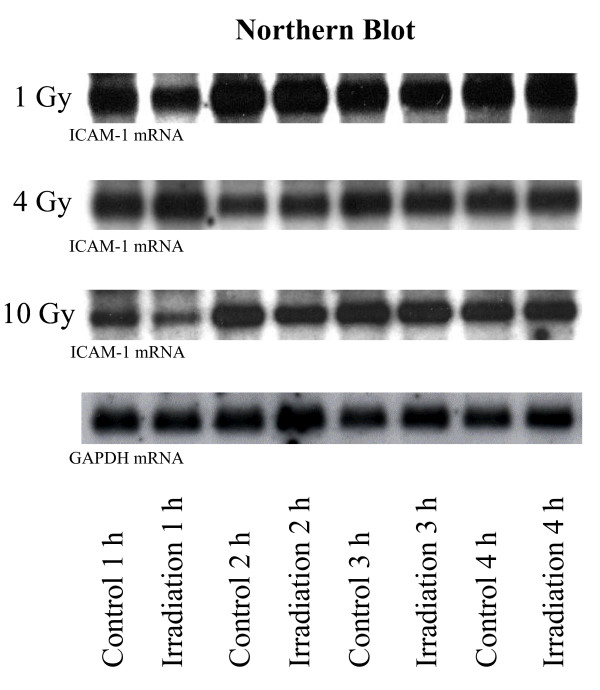
Northern blots of ICAM-1 expression 1 h, 2 h, 3 h, and 4 h after low dose irradiation of human MC with doses of 1 Gy, 4 Gy, and 10 Gy. Expression of ICAM-1 in quiescent MC was not affected by low dose irradiation, GAPDH was used as control.

**Figure 4:**
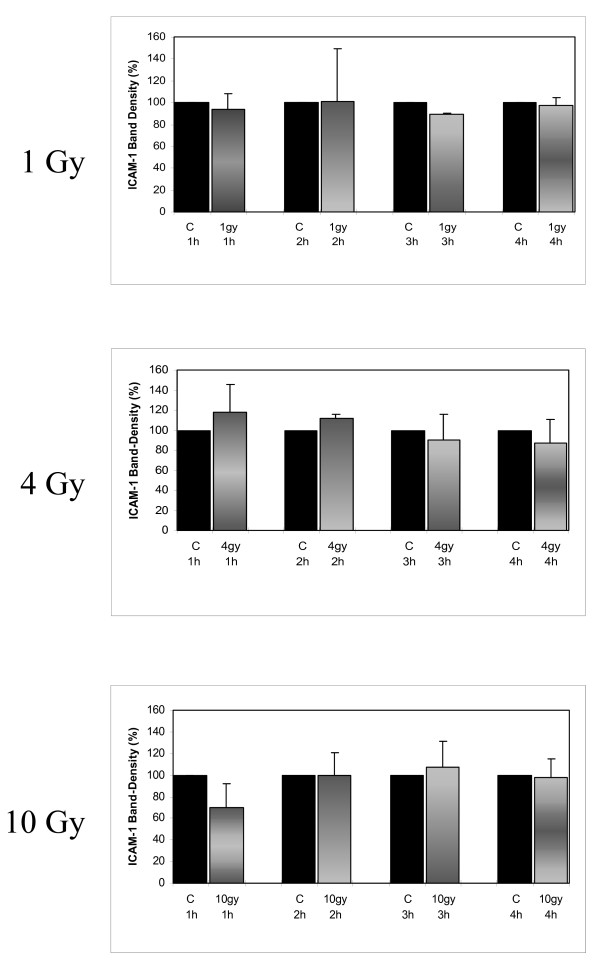
Relative band density of Northern blots of ICAM-1 expression 1 h, 2 h, 3 h, and 4 h after low dose irradiation of human MC with doses of 1 Gy, 4 Gy, and 10 Gy. Expression of ICAM-1 in quiescent MC was not affected by low dose irradiation.

Band density of mRNA ICAM-1 was analyzed 1 h, 2 h, 3 h, and 4 h after irradiation of quiescent MC with 1 Gy, 4 Gy, and 10 Gy. Apart from two exceptions (3 h/1 Gy and 2 h/4 Gy) no significant, dose dependent inhibition of ICAM-1 expression was achieved (Fig. 3 and 4).

1 h, 2 h, 3 h, and 4 h after irradiation with 1 Gy band density of mRNA ICAM-1 was 93.5 ± 15%, 100.8 ± 49%, 89.1 ± 2%, and 97.2 ± 7% in comparison to untreated controls. Statistical significance (p < 0.01) was achieved 3 h after irradiation with 1 Gy. Furthermore, 1 h, 2 h, 3 h, and 4 h after irradiation with 4 Gy band densities of mRNA ICAM-1 were 117.6 ± 28%, 111.6 ± 4%, 90.4 ± 26%, and 87.4 ± 23% in comparison to untreated controls, reaching statistical significance 2 h after irradiation with 4 Gy (p < 0.05). Finally, irradiation with 10 Gy resulted in mRNA ICAM-1 band densities of 69.6 ± 22%, 99.9 ± 21%, 107.2 ± 24% and 97.6 ± 17% in comparison to untreated controls, statistical significance was not reached. Expression of mRNA GAPDH after irradiation with 1, 4, 10 Gy was identical with un-radiated controls (Fig. 3).

## Discussion

The present study investigates the effect of low dose irradiation (LDI) on proliferation of SMC and expression of ICAM-1 in MC. We came to three basic conclusions after completing the present study. First, irradiation with one Gy caused both inhibitory (day 10 and day 15) and stimulatory effects (day 20) on HPSMC proliferation. Second, dose dependent antiproliferative effects were detected after irradiation of HPSMC with 4 Gy and 10 Gy. Third, expression of ICAM-1 in quiescent MC was not affected.

The pathophysiology of the edge effect is incompletely understood. Current discussions point out that the edge effect may be the result of vessel wall injury, low-dose radiation at the edges of the irradiated area or a combination of both effects [5,6]. Syeda et al. [7] studied the baseline angiograms of 112 vessels in 109 patients with in stent restenosis undergoing reintervention followed by intracoronary irradiation and suggested a safety margin of 10 mm per vessel in order to prevent edge restenosis.

It remains to be investigated whether irradiation of the margins is really able to solve the problem [5,9,10]. It is not yet entirely clear whether the subtherapeutic levels of radiation in uninjured segements will not cause an inhibitory and later stimulatory effect. The group of Powers et al. [9] studied the effect of irradiation with 10 Gy – 55 Gy in noninjured aortic segments of dogs. Two and five years after irradiation neointimal proliferation was inhibited with irradiation doses > 20 Gy but stimulated in segments with doses < 20 Gy. If HPSMC and quiescent MC react in the same manner there is a problem, extending the irradiation area or increasing the irradiation dose would merely relocate the restenotic zone further from the main irradiation area.

The term LDI is not exactly defined. With the Beta-Cath system as used by Sabaté et al. [15] the prescribed dose ranged between 12 Gy and 20 Gy at 2 mm from the source axis. The dose received 1, 2, 3, 4, and 5 mm from the 100% isodose is 86% (10.3 – 17.2 Gy), 60% (7.2 – 12 Gy), 30% (3.6 – 6 Gy), 13% (1.56 – 2.6 Gy), and 5% (0.6 – 1 Gy). In the current study the effect of LDI with 1 Gy, 4 Gy, and 10 Gy on proliferation of SMC and expression of ICAM-1 in MC was investigated, representing the situation 5 mm, 3 mm, and 2 mm of the 100% isodose.

After irradiation of HPSMC with 1 Gy both inhibitory and stimulatory effects on cell proliferation had been detected. At day 10 and day 15 after irradiation a small but significant inhibitory effect was detected, at day 20 after irradiation a significant stimulatory effect was found. It has been reported by Eidus et al. [16] that irradiation with 2 Gy potentiates cellular metabolic acitivities and immunological responses in various cells of mesodermal origin [17]. Furthermore, experimental studies of endovascular brachytherapy have shown that relatively low doses of irradiation (10 Gy) caused a paradoxical increase in tissue response [18], whereas higher doses proved to be antiproliferative. The switch from the inhibitory effect to the stimulatory effect as demonstrated in the current study after irradiation with 1 Gy is of special interest. It may be speculated that after LDI the irradiation damage of the cells causes both inhibitory and stimulatory effects. The net effect of these two divergent forces is responsible for the effect detected in vitro: an inhibitory net effect in the first period followed by a stimulatory effect in the second period. If this hypothesis is transferred to the results found after irradiation with 4 Gy and 10 Gy, it is possible that the period of 20 days was too short to detect the second phase of stimulation.

Some authors are advocating that the key cells responsible for restenosis are monocytes/macrophages, the same cells that initiates atherosclerotic plaques [review, [13]]. Using the paradigm of the monocyte-derived macrophage as the pivotal, central player, radiation and LDI could affect further upstream in the chain of events. Because these recruited MC are responsible for triggering the cytokine and chemokine cascades, LDI of quiescent MC in the uninjured but atherosclerotic vessel wall could potentially initiate adhesion of circulating MC and/or trigger subsequent proliferative activity of SMC. Recently the role of ICAM-1 in the pathogenesis of experimental radiation induced inflammation was studied [19]. Molla et al. [19] demonstrated that leukocyte adhesion 24 h after abdominal radiation of mice with 10 Gy is significantly reduced in ICAM-1 (-/-) mice or after treatment with ICAM-1 antibodies.

In the current study neither stimulatory nor inhibitory effects of LDI on ICAM-1 expression in MC were detected. These data are in accordance with reports of Hildebrandt et al. [20] describing the effect of LDI (0.3 Gy – 5 Gy) on ICAM-1 expression in HUVEC. The difficulties in the interpretation of data with LDI can be demonstrated with two respectively three exceptions that occurred both in the current study and in the report of Hildebrandt et al. [20]. A significant inhibition of ICAM-1 expression in MC was described in the present study 3 h after irradiation with 1 Gy and a significant stimulation was found 2 h after irradiation with 4 Gy. In the study of Hildebrandt et al. [20] in HUVEC a significant inhibition of ICAM-1 expression was reported 4 h after irradiation with 0.3 Gy and 1 Gy and a significant stimulation was found 6 h after irradiation with 5 Gy. As discussed biological deviations may explain the effects. It is nevertheless surprising that two independent groups describe almost the same exceptions. If the explanation of the effects as biological deviations is correct the data do not support the hyothesis that LDI of quiescent MC in the uninjured segments close to the injury may stimulate the genesis of the edge effect via expression of ICAM-1.

### Limitations of the study

It has been reported that differences in proliferation rates [11], cell constitution and plaque characteristics exist not only between primary stenosing and restenosing plaque tissue but as well within the group of primary stenosing plaque material [21], depending on the status of the lesion. In order to minimize these influences pooled HCPSMC of various donors from both sexes were used for cell proliferation studies.

Moreover, cell number is influenced by proliferating and dying cells. In future studies the effect of LDI on apoptosis should be studied.

Due to the fact that proliferation of human SMC can be influenced by culture conditions [22], cell density might have had an impact on the effect of LDI. Björkerud [23] and Ikedo et al. [24] reported that, depending on culture conditions, TGF-β can either promote or inhibit proliferation of SMC in vitro.

Although the study design tried to imitate the complex situation of LDI in uninjured but atherosclerotic segments as close as possible, the in vivo situation is much more complex. If the interpretation is correct that a first inhibitory phase is followed by a second stimulatory phase of cell proliferation, the investigated period of 20 days was too short to detect the stimulatory phase in the group treated with 4 Gy and 10 Gy. However, with the applied seeding concentration of 5000 cells/cm^2^, a period of 20 days is the maximal duration of cell proliferation before confluency is reached.

## Conclusion

Many authors suggest to irradiate the margins of the main intervention area to minimize or avoid the edge effect [7,8]. The current data indicate that LDI of HPSMC inhibits cell proliferation in the short run but may stimulate cell proliferation in the long run. Irradiation of edges may therefore not eliminate but merely postpone the problem of edge restenosis.

## Competing interests

The author(s) declare that they have no competing interests.

## Authors' contributions

All authors read and approved the final manuscript. RV, RB, AH, and VH designed the study, RV wrote the manuscript. JH and RB carried out the Northern blot studies, cell proliferation studies were done by RB and TH.

## Pre-publication history

The pre-publication history for this paper can be accessed here:


